# Effects of Dried Plum Supplementation on Bone Metabolism in Adult C57BL/6 Male Mice

**DOI:** 10.1007/s00223-013-9819-2

**Published:** 2013-12-20

**Authors:** B. J. Smith, J. L. Graef, T. J. Wronski, E Rendina, A. A. Williams, K. A. Clark, S. L. Clarke, E. A. Lucas, B. P. Halloran

**Affiliations:** 1 Department of Nutritional Sciences, 420 College of Human Sciences, Oklahoma State University, Stillwater, OK USA; 2 Department of Physiological Sciences, College of Veterinary Medicine, University of Florida, Gainesville, FL USA; 3 Division of Endocrinology, Department of Medicine, Veterans Affairs Medical Center, University of California at San Francisco, San Francisco, CA USA

**Keywords:** Aging, Osteoporosis, Oxidative stress, Plum, Skeleton

## Abstract

Dietary supplementation of dried plum (DP) prevents bone loss and restores bone mass in osteopenic animal models. This study was designed to determine the effects of DP supplementation on bone metabolic activity over time using adult (6-month-old) male C57BL/6 mice (*n* = 40) receiving control (CON = AIN93 M) or CON+DP 25 % (w/w) diets for 4 or 12 weeks. After 4 weeks of treatment, animals consuming the DP diet had a higher whole-body bone mineral density, vertebral trabecular bone volume (BV/TV), and femoral cortical thickness compared to the CON animals. In the distal metaphysis of the femur, BV/TV was increased in the DP-treated animals, but only after 12 weeks. Bone histomorphometric analyses revealed that DP decreased osteoblast surface (67 %) and osteoclast surface (62 %) at 4 weeks, but these surfaces normalized to the CON animals by 12 weeks. Coincident with these changes, the mineralizing surface (MS/BS) and cancellous bone formation rate (BFR/BS) were reduced at 4 weeks in the DP group compared to the CON, but by 12 weeks of DP supplementation, BFR/BS (~twofold) and MS/BS (~1.7-fold) tended to be increased (*p* < 0.10). The relative abundance of RNA for key regulators of osteoblast and osteoclast differentiation and indicators of osteoblast activity were reduced in the DP group at 4 weeks with no difference between groups at 12 weeks. These results indicate that supplementing the diet with DP initially suppressed cancellous bone turnover, but a biphasic response occurs over time, resulting in a positive effect on bone mass and structure.

## Introduction

Age-related osteoporosis is a significant public health problem for both men and women. Projected estimates indicate that by 2020, 50 % of women and 25 % of men over the age of 50 years will experience an osteoporotic fracture during their lifetime [1]. Fractures not only compromise activities of daily living but also increase overall mortality, especially in the 12 months immediately after a hip fracture [2]. The adult skeleton undergoes bone remodeling, and optimal bone health requires a metabolic balance between bone formation by osteoblasts and bone resorption by osteoclasts. In age-related bone loss, osteoclast and osteoblast activity becomes uncoupled, resulting in a reduced rate of bone formation relative to the rate of bone degradation [3]. This uncoupling of bone cell activity has been attributed to multiple factors, including increased prevalence of nutritional insufficiencies (e.g., calcium, vitamin D, and protein), age-related alterations in endocrine status (e.g., decreased estrogen and insulinlike growth factor or IGF-1), decreased physical activity, increased use of medications that affect bone, and bone cell senescence [4, 5].

Current treatment strategies for osteoporosis consist of several different options with antiresorptive and anabolic properties [3]. The available antiresorptive therapies include bisphosphonates, hormone replacement therapy, denosumab, and selective estrogen receptor modulators, with bisphosphonates being the most commonly prescribed class of drugs [3]. Intermittent parathyroid hormone therapy is the only U.S. Food and Drug Administration-approved anabolic agent for treatment of patients with severe osteoporosis [3, 6]. In many countries, strontium ranelate is considered an attractive treatment option that reputedly has both anabolic and antiresorptive properties, but it has not received approval for use in the United States [3, 7]. Despite these significant advances in pharmacological options for the treatment of osteoporosis over the past two decades, issues with patient compliance, adverse effects, cost, and long-term efficacy have remained [3]. Another approach, nutritional supplementation, has typically focused on calcium and vitamin D. The combination of calcium and vitamin D has been promoted for reducing the risk of fracture primarily by reducing bone resorption. However, recent recommendations by the U.S. Preventive Services Task Force have brought the use of calcium and vitamin D supplementation (≤1,000 mg Ca^2+^ and ≤400 IU D3) as a primary fracture prevention strategy into question [8]. Therefore, the search has continued for effective, low-cost treatment strategies with fewer side effects.

Among the dietary approaches that have been considered, dried plum (DP) has demonstrated unique biological activity that is based on its ability to prevent and reverse bone loss. Studies in animal models of gonadal hormone deficiency have provided important insight into this response [9–12]. For example, DP supplementation prevented bone loss associated with ovarian hormone deficiency in C57BL/6 mice [12] and has been demonstrated to be more effective than other fruits in its ability to restore bone in osteopenic ovariectomized mice [11]. In male osteoporosis, DP supplementation was shown to prevent orchidectomy-induced bone loss by Sprague–Dawley rats [10], and in a subsequent study, DP restored bone in this animal model [9]. Interestingly, DPs capacity to restore trabecular bone in the osteopenic animals was similar to intermittent parathyroid hormone treatment [9]. Findings from these studies demonstrated that DP suppressed bone resorption as indicated by systemic markers of bone resorption (e.g., urinary deoxypyridinoline excretion and serum pyridinoline crosslinks), which was mediated by the down-regulation of osteoclastogenesis through the suppression of receptor activator for NF-kappa B ligand (RANKL) signaling [9, 10]. In several studies, the decrease in bone resorption coincided with an increase in serum IGF-I, but the extent to which bone formation is altered remains unclear [10, 13]. Clinical studies have also supported the efficacy of DP supplementation on bone health [13, 14].

In addition to the effects of DP on bone in gonadal hormone deficiency, it has also been shown to be effective in improving bone parameters in age-related bone loss. Recently we reported that male adult and old C57BL/6J mice (i.e., 6 and 18 months of age) fed a DP-supplemented diet for 6 months experienced a 48 and 34 % increase, respectively, in trabecular bone volume in the distal femoral metaphysis [15]. On the basis of in vivo microcomputed tomography (microCT) data from that study, the majority of the response to DP occurred during the first 3 months of the study, but the mechanism by which DP exerted these beneficial effects remained unclear. Until now, to our knowledge, no studies have examined the effects of DP supplementation on age-related bone loss at early time points. This study was designed to gain a better understanding of the bone metabolic changes occurring in response to DP supplementation during the first 12 weeks of treatment and the mechanisms involved in a murine model of age-related osteoporosis. The hypothesis to be tested was that DP supplementation would increase indices of bone formation and suppress resorption at 4 and 12 weeks, which would result in improved bone structural properties.

## Materials and Methods

### Animal Care, Diet, and Necropsy

Six-month-old male C57BL/6 mice (*n* = 40; Charles River Laboratories, Wilmington, MA, USA) were assigned to AIN-93M control diet or control diet with 25 % (w/w) DP for 4 or 12 weeks (*n* = 10/group). The DP powder was provided by the California Dried Plum Board. The detailed formulation of the DP diet has been published previously [12]. The mice were allowed to acclimate to an environmentally controlled room on a 12-h light/dark cycle in an animal care facility for 3 days before beginning consumption of the experimental diets. The mice were group housed (4–5 mice/cage) on wire-bottom grids to minimize coprophagic activity and had free access to deionized water throughout the treatment period. Body weights and food consumption were monitored weekly.

After the 4- or 12-week treatment period, the mice were fasted for 6 h before anesthetization with a ketamine (70 mg/kg body weight) and xylazine (3 mg/kg body weight) mixture. Whole body dual-energy X-ray absorptiometry scans (DXA; Lunar PIXImus, Madison, WI, USA) were performed. The animals were then bled from the carotid artery, and blood was collected and allowed to coagulate for 15–20 min before separation of serum by centrifugation (3,000 rpm × 20 min). The serum was aliquoted and stored at −80 °C until analyses were performed. One femur from each animal was flushed with ice-cold PBS, and RNA was extracted from the bone marrow and hard tissue separately. The other femur was collected and stored in PBF for 48 h before being transferred to and stored in 70 % ethanol at 4 °C for X-ray and histomorphometric analyses. The spine was stored at −20 °C until DXA and microCT analyses were performed. All experimental procedures were approved by the Animal Care and Use Committee at Oklahoma State University.

### Whole-Body and Vertebral DXA Absorptiometry Analyses

Whole-body and vertebral DXA scans were performed to assess bone mineral content (BMC), bone mineral area (BMA), and bone mineral density (BMD). Whole-body scans were completed on anesthetized animals before necropsy. DXA analyses of the spine were completed by scanning the excised spine and analyzing the fourth lumbar vertebra.

### Biochemical Markers of Bone Metabolism

Systemic markers of bone formation and bone resorption were analyzed. Serum procollagen type 1 amino-terminal propeptide (P1NP), which is released from procollagen during collagen synthesis and is considered an indicator of bone formation, was assessed using a commercially available ELISA kit (IDS, Scottsdale, AZ). The sensitivity of the assay is 0.7 ng/ml, and the inter- and intra-assay variability was 9.2 and 6.4 %, respectively. In addition, serum levels of pyridinoline, a bone resorption marker released during the breakdown of hydroxy lysyl crosslinks in collagen, were also assessed using an ELISA kit (MyBioSource, San Diego, CA, USA). The sensitivity of the assay is <2 pg/ml, and the inter- and intra-assay variability was <10 and <8 %, respectively.

### Glutathione Peroxidase Activity in Serum

Glutathione peroxidase activity, an indicator of antioxidant capacity, was measured in the serum using a commercially available assay kit and following the manufacturer’s guidelines (Cayman Chemical Company, Ann Arbor, MI, USA). The sensitivity of this dynamic, multimeasurement assay was 50–344 nmol/min/ml, which represents an absorbance decrease of 0.02–0.135 per minute. The inter- and intra-assay variability was 7.2 and 5.7 %, respectively.

### Trabecular and Cortical Bone Microarchitecture

MicroCT scans (SCANCO Medical AG, Switzerland) were performed on the fourth lumbar vertebra and the distal femoral metaphysis to assess the microstructure of the trabecular and cortical compartments of bone. A region (1.8 mm) within the distal femoral metaphysis was scanned at a resolution of 2,048 × 2,048 pixels, and the volume of interest (VOI = 0.9 mm) was analyzed by placing semiautomatic contours to evaluate the secondary spongiosa. For analysis of vertebral trabecular bone, the lumbar vertebral body was scanned at a resolution of 1,024 × 1,024 pixels. Secondary spongiosa within an ~4.2 mm VOI located between cranial and caudal growth plates was analyzed. At both sites, trabecular microarchitecture was analyzed, including trabecular bone volume expressed as a percentage of total volume (BV/TV), trabecular number (TbN), trabecular thickness (TbTh), trabecular separation, connectivity, and the structural model index.

MicroCT scans were also performed on the mid-diaphysis of the femur for cortical bone analyses. An ~0.3-mm region at the midshaft was scanned and analyzed for cortical parameters. Cortical thickness, cortical area, and porosity as well as medullary area were determined within the VOI.

### Static and Dynamic Bone Histomorphometry

Bone histomorphometry was performed in the secondary spongiosa within the distal femur metaphysis. Calcein (10 mg/kg, pH 7.4) was injected 7 and 2 days before the end of the study to allow for the determination of dynamic bone histomorphometric indices. Excised femurs were fixed in 10 % phosphate-buffered formalin, dehydrated in graded ethanol solutions and xylene, and then embedded (undecalcified) in methyl methacrylate. Von Kossa staining with a tetrachrome counterstain was utilized for measurement of osteoblast and osteoclast surfaces in longitudinal sections (4 μm thick) of the distal femoral metaphysis. Unstained 8-μm-thick longitudinal sections were used to determine dynamic indices of cancellous bone formation, including mineralizing surface (MS/BS), mineral apposition rate (MAR), and surface-based bone formation rate (BFR/BS). All measurements were made with the OsteoMeasure System (Osteometrics, Atlanta, GA, USA).

### Gene Expression Analysis in Bone Marrow and Flushed Femur Using Real-time PCR

Total RNA was extracted from the bone marrow and the flushed femur specimens using Trizol reagent (Invitrogen, Rockville, MD, USA) and the protocol provided by the manufacturer. Briefly, bone marrow samples from the flushed femur were centrifuged at 2,000×*g* for 5 min, the supernatant was decanted and RNA extraction with Trizol was performed. The flushed femur was milled in a liquid nitrogen–containing freezer mill (Spex 6770 Freezer Mill) before RNA extraction using Trizol. The concentration and quality of the RNA were determined by measuring OD at 260 and 280 nm with a Nanodrop Spectrophotometer (Rockland, DE, USA) and electrophoretic analysis on an agarose gel. Total RNA (2 μg) was then pretreated with DNase I and subjected to reverse transcription (Superscript II, Invitrogen, Carlsbad, CA, USA). cDNA (50 ng) was used for each qRT-PCR reaction, and reactions were evaluated in duplicate using SYBR green chemistry (Roche, Penzberg, Germany) on Applied Biosystems 7900HT Fast Real-Time PCR System (Foster City, CA, USA). Gene-specific primers related to osteoblast and osteoclast differentiation and activity were used to amplify sequences of interest (Table 1) to determine relative abundance. Expression levels of each gene were normalized to the reference gene *Cyclo* in both the bone marrow and the flushed femur samples. Comparison of the quantification cycle (Cq) was done to determine differences in gene expression between dietary treatment groups at each time point.

**Table 1:** Primer sequences for qPCR

Transcript	Sequence (5′–3′)
BMP4	F: GCC GAG CCA ACA CTG TGA
	R: TGG TCC CTG GGA TGT TCT C
Runx2	F: CGA CAG TCC CAA CTT CCT GT
	R: CGG TAA CCA CAG TCC CAT CT
Osterix (Osx)	F: GAA GTT CAC CTG CCT GCT CTG T
	R: CGT GGG TGC GCT GAT GT
ALP	F: GGT ATG GGC GTC TCC ACA GT
	R: GCC CGT GTT GTG GTG TAG CT
Col1a1	F: CGT CTG GTT TGG AGA GAG CAT
	R: GGT CAG CTG GAT AGC GAC ATC
Ocn	F: CTG ACA AAG CCT TCA TGT CCA C
	R: GCG CCG GAG TCT GTT CAC TA
RANKL	F: TCT GCA GCA TCG CTC TGT TC
	R: AGC AGT GAG TGC TGT CTT CTG ATA TT
OPG	F: TCC CGA GGA CCA CAA TGA AC
	R: TGG GTT GTC CAT TCA ATG ATG T
Nfatc1	F: GCG AAG CCC AAG TCT CTT TCC
	R: GTA TGG ACC AGA ATG TGA
Ctsk	F: GCA GGA TGT GGG TGT TCA AGT
	R: TCC GGA GAC AGA GCA AAG CT

### Statistical Analyses

Statistical analyses were performed by SAS version 9.2 (SAS, Cary, NC, USA). Analysis of treatment effects at each time point were conducted by Student’s *t* tests, with *p* < 0.05 considered statistically significant. Data are presented as means ± standard errors (SE) with *n* = 5–10 mice/group.

## Results

### Body Weights, Body Composition, and Food Intake

The mice in both treatment groups continued to experience an increase in body weight throughout the study, and no differences occurred in food intake (Table 2). Body mass did not differ between treatment groups at 4 weeks, but by the 12-week time point, the DP group had a lower body mass than the controls (*p* < 0.05). On the basis of analysis of body composition, the DP group had a significantly lower fat mass compared to the control group, but no differences were detected in lean mass (Table 2).

**Table 2:** Food intake, body weight and composition, and DXA analyses

Characteristic	4-week CON	4-week DP	*p*-value	12-week CON	12-week DP	*p*-value
Food intake (g/mouse/day)	6.4 ± 0.03	6.4 ± 0.11	0.5394	6.3 ± 0.06	6.3 ± 0.05	0.2349
Body weight (g)	35.7 ± 0.8	35.4 ± 0.8	0.4211	42.4 ± 0.5	38.2 ± 0.7*	0.0003
Lean mass (g)	25.1 ± 0.5	24.9 ± 0.4	0.6951	26.1 ± 0.4	25.1 ± 0.5	0.1183
Fat mass (g)	9.1 ± 0.6	9.2 ± 0.8	0.8971	15.0 ± 0.7	11.4 ± 0.8*	0.0029
Fat (%)	26.4 ± 1.3	26.9 ± 1.7	0.9838	36.4 ± 1.3	31.1 ± 1.7*	0.0240
Whole body						
BMA (cm^2^)	11.077 ± 0.206	11.247 ± 0.223	0.5827	10.094 ± 0.217	11.070 ± 0.324*	0.0300
BMC (mg)	569.1 ± 16.7	605.2 ± 16.0	0.1271	514.5 ± 11.5	595.9 ± 14.1*	0.0006
BMD (mg/cm^2^)	51.3 ± 0.06	53.9 ± 0.06*	0.0086	50.3 ± 0.9	53.5 ± 0.8*	0.0174
Vertebra						
BMA (cm^2^)	1.027 ± 0.019	1.024 ± 0.017	0.9515	1.130 ± 0.03	1.108 ± 0.025	0.5292
BMC (mg)	46.2 ± 0.9	50.6 ± 1.4*	0.0227	50.5 ± 1.2	55.9 ± 1.7*	0.0175
BMD (mg/cm^2^)	45.2 ± 0.4	49.4 ± 1.1*	0.0029	44.7 ± 0.8	50.5 ± 0.9*	0.0002

*DXA* dual-energy X-ray absorptiometry, *CON* control, *DP* dried plum, *BMD* bone mineral density, *BMC* bone mineral content, *BMA* bone mineral area

* Treatment group statistically different from control at given time point, *p* < 0.05; *n* = 9–10 mice/group

### Whole-Body and Vertebral DXA Analyses

Whole-body and vertebral BMD were significantly greater in the DP group compared to the control group at both 4 and 12 weeks (Table 2). The higher BMD with DP treatment coincided with a significantly greater BMC of the vertebra (*p* < 0.05) at 4 weeks and in both the vertebra and whole body at 12 weeks. Whole-body BMA was higher (*p* < 0.05) in the DP group at 12 weeks.

### Trabecular and Cortical Bone Microarchitectural Properties

To examine the effect of DP supplementation on the cortical and trabecular components of bone, microCT analyses were completed on both the fourth lumbar vertebra and the femur. Similar to the positive effects observed on BMD, microarchitectural analyses of vertebral trabecular bone indicated a positive effect of DP as early as 4 weeks, as BV/TV was greater in the DP group compared to control (*p* < 0.05), but there were no statistically significant differences in TbN or TbTh (Table 3). The response of the trabecular bone in the lumbar vertebra was more rapid than the distal femur metaphysis as there were no differences between treatment groups at 4 weeks in any of the trabecular parameters in the femur (Table 3). After 12 weeks of treatment, the DP group had greater BV/TV at both the vertebral and the distal femoral sites. The alterations in the trabecular bone at 12 weeks in response to DP were characterized by a higher TbN and correspondingly reduced trabecular separation compared to the control group. Animals receiving the DP diet showed a trend (*p* = 0.06) toward increased TbTh in the distal femoral metaphysis at 12 weeks, but there were no statistically significant improvements in TbTh in the distal femur or vertebral body at either time point.

**Table 3:** MicroCT analyses of trabecular and cortical bone microarchitecture

Site	4-wk CON	4-wk DP	*p*-value	12-wk CON	12-wk DP	*p*-value
Lumbar vertebra						
BV/TV (%)	14.1 ± 0.01	17.6 ± 0.01*	0.0185	13.7 ± 0.01	18.2 ± 0.01*	0.0198
Trabecular number (1/mm^2^)	4.20 ± 0.10	4.44 ± 0.09	0.1039	4.00 ± 0.14	4.50 ± 0.12*	0.0138
Trabecular thickness (μm)	47.8 ± 0.9	49.2 ± 1.2	0.3717	47.9 ± 1.0	47.9 ± 1.2	1.000
Trabecular separation (μm)	232.5 ± 6.3	218.4 ± 5.7	0.1130	245.0 ± 10.0	214.3 ± 6.7*	0.0194
Conn Dens (1/mm^3^)	89.4 ± 7.3	117.2 ± 8.6*	0.0260	78.5 ± 9.7	136.7 ± 10.9*	0.0009
SMI	2.03 ± 0.08	1.59 ± 0.13*	0.0122	2.02 ± 0.12	1.50 ± 0.13*	0.0109
Distal femur metaphysis						
BV/TV (%)	9.9 ± 0.6	11.6 ± 1.1	0.1827	6.7 ± 0.5	11.0 ± 0.7*	0.0003
Trabecular number (1/mm^2^)	3.48 ± 0.14	3.71 ± 0.05	0.1421	2.01 ± 0.12	3.02 ± 0.21*	0.0007
Trabecular thickness (μm)	44.6 ± 1.9	46.8 ± 2.7	0.5349	33.3 ± 1.1	37.2 ± 1.8	0.0602
Trabecular separation (μm)	279.7 ± 12.5	256.3 ± 4.4	0.0963	482.5 ± 33.8	307.7 ± 23.1*	0.0025
Conn Dens (1/mm^3^)	80.4 ± 10.5	84.3 ± 5.0	0.7427	37.5 ± 4.9	66.8 ± 7.5*	0.0105
SMI	1.82 ± 0.10	1.87 ± 0.10	0.7580	2.51 ± 0.06	1.91 ± 0.06*	<0.0001
Femur mid-diaphysis						
Cortical thickness (mm)	0.201 ± 0.003	0.211 ± 0.002*	0.0267	0.190 ± 0.004	0.213 ± 0.003	0.0026
Cortical area (mm^2^)	0.997 ± 0.014	1.042 ± 0.014	0.5051	0.949 ± 0.020	1.042 ± 0.014	0.0044
Medullary area (μm^2^)	10.13 ± 0.32	10.10 ± 0.40	0.9880	11.56 ± 0.35	12.13 ± 0.82	0.5328
Porosity (%)	0.98 ± 0.035	1.00 ± 0.036	0.7575	1.23 ± 0.057	1.109 ± 0.034	0.7599

*CON* control, *DP* dried plum, *BV/TV* trabecular bone volume, *Conn Dens* connectivity density, *SMI* structural model index

* Treatment group statistically different from control at given time point, *p* < 0.05; *n* = 9–10 mice/group

Nonmorphometric analyses of trabecular bone also indicated a positive effect of DP (Table 3). The connectivity of trabecular bone was greater in the DP group at 4 weeks in the vertebra and in both the vertebra and femur at 12 weeks (*p* < 0.05). A lower structural model index, which is associated with a structure that has improved bone biomechanical properties, was observed with DP supplementation in the vertebral body at 4 and 12 weeks and in the femur at 12 weeks. These findings indicate that in the DP-treated animals, the trabeculae had a more platelike structural arrangement (Table 3).

Cortical bone analyses of the mid-diaphysis of the femur showed a significantly greater cortical thickness in the DP group compared to control at both the 4- and 12-week time points (Table 3). There were no differences between treatment groups in cortical area, medullary area, or porosity at 4 weeks, but cortical area was increased (*p* < 0.01) with DP by the 12th week.

### Biochemical Markers of Bone Metabolism

**Fig. 1:**
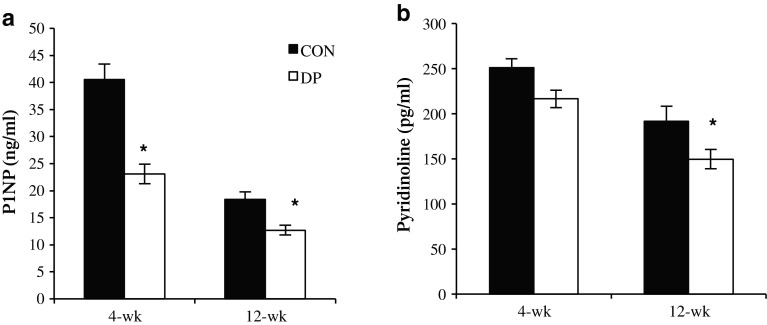
DP supplementation alters serum markers of bone metabolism at 4 and 12 weeks: **a** procollagen type 1 amino terminal propeptide (P1NP) and **b** pyridinoline crosslinks. *Bars* represent the mean ± SE. *t* tests were performed at each time point to compare DP treatment groups to their respective controls. *p < 0.05; *n* = 9–10 mice/group

Despite an increase in whole-body BMD observed, systemic markers of bone metabolism indicated a suppression of bone formation at 4 weeks in the DP group. Serum P1NP was reduced by 43 % in the DP group compared to control at this time point (Fig. 1a). Interestingly, P1NP remained suppressed compared to the controls at 12 weeks (*p* < 0.05), but it was apparent that the control P1NP was ~50 % lower than at 4 weeks. There was no difference in serum pyridinoline between the DP and control groups at 4 weeks, but serum pyridinoline was significantly lower in the DP group at 12 weeks, indicating a decrease in bone resorption (Fig. 1b).

### Glutathione Peroxidase Activity in Serum

**Fig. 2:**
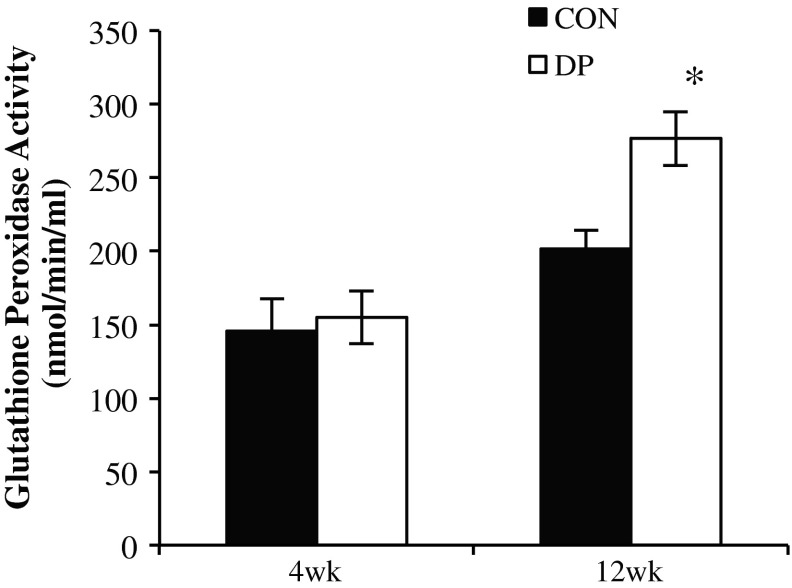
Alterations in serum glutathione peroxidase activity in response to 4 and 12 weeks of DP treatment. Bars represent the mean ± SE; *t* tests were performed at each time point to compare DP treatment groups to their respective controls. *p < 0.05; *n* = 9–10 mice/group

To determine whether the skeletal improvements in the DP group were linked to greater antioxidant capacity, glutathione peroxidase activity in the serum was examined. Glutathione peroxidase activity was not different between groups at 4 weeks (Fig. 2). However, the DP group had significantly higher glutathione peroxidase activity than the control at 12 weeks, suggesting a greater antioxidant capacity as a result of DP supplementation (*p* < 0.01).

### Bone Histomorphometry

**Fig. 3:**
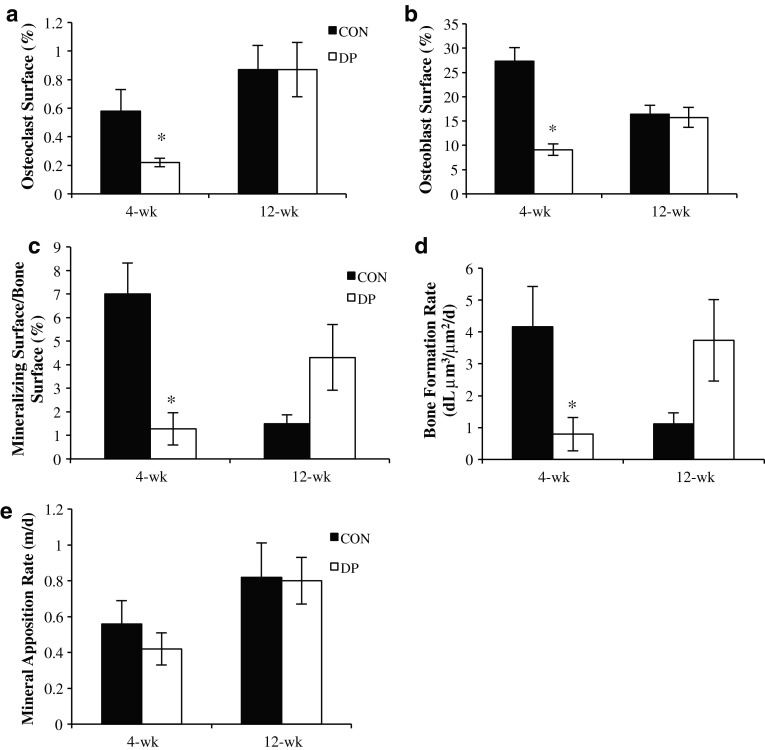
Effects of DP treatment at 4 and 12 weeks on trabecular bone histomorphometric indices of the distal femoral metaphysis: **a** osteoclast surface, **b** osteoblast surface, **c** mineralizing surface, **d** bone formation rate, and **e** mineral apposition rate. Values presented are mean ± SE, and statistical analyses consisted of *t* tests to compare treatment groups at each time point. *DP treatment group is different from control at given time point, *p* < 0.05; *n* = 5–10 mice/group

At the end of 4 weeks, the osteoclast (*p* < 0.05) and osteoblast (*p* < 0.001) surfaces of the distal femur metaphysis were decreased in the DP group compared to control. The magnitude of this effect was a 62 % reduction in the osteoclast surface (Fig. 3a) and a 68 % reduction in the osteoblast surface (Fig. 3b). Dynamic histomorphometry showed a suppression of MS/BS (*p* < 0.01) (Fig. 3c) and BFR/BS (*p* < 0.05) (Fig. 3d) after 4 weeks of DP supplementation. Interestingly, after 12 weeks of DP supplementation, cancellous BFR/BS and MS/BS rebounded, with the DP group trending toward statistically greater BFR/BS (*p* = 0.08) and MSBS (*p* = 0.08) than the control group. Both osteoclast and osteoblast surfaces in the DP group had normalized to the level of the control group by 12 weeks. MAR was not different between treatment groups at either time point (Fig. 3e).

### Gene Expression Markers of Osteoblast and Osteoclast Differentiation and Activity

**Fig. 4:**
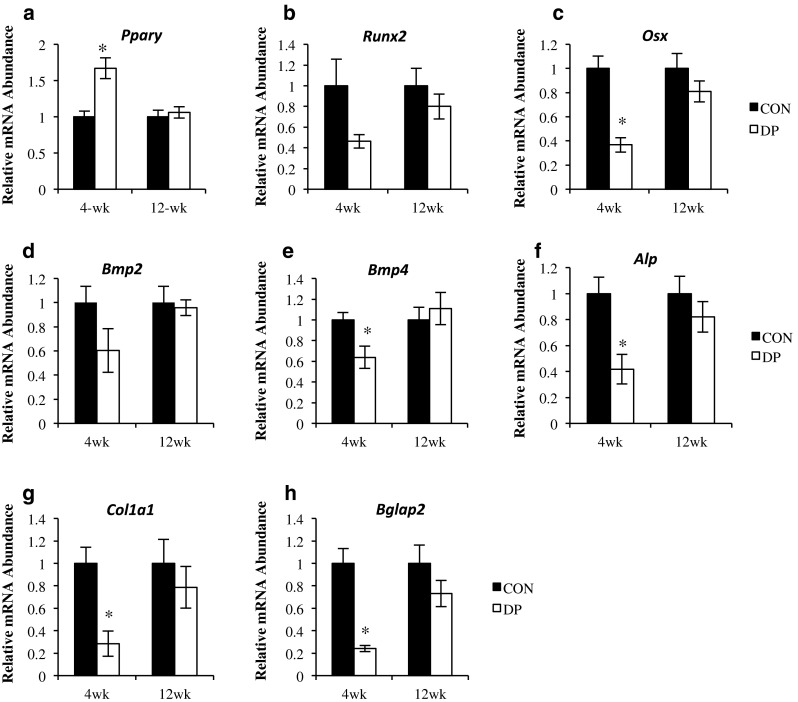
Alterations in gene expression related to osteoblast differentiation and activity. The relative mRNA abundance of **a** peroxisome proliferator-activated receptor gamma (*Pparγ*), **b** runt-related transcription factor 2 (*Runx2*), **c** osterix (*Osx*), **d** bone morphogenetic protein 2 (*Bmp2*), **e** bone morphogenetic protein 4 (*Bmp4*), **f** alkaline phosphatase (*Alp*), **g** collagen type 1, alpha 1 (*Col1a1*), and **h** osteocalcin (*Bglap2*) are shown after 4 and 12 weeks of DP supplementation. *DP treatment group is different from control at given time point, *p* < 0.05; *n* = 5–6 mice/group

Analysis of qRT-PCR data also indicated that osteoblast differentiation was suppressed at 4 weeks in the DP group (Fig. 4). The relative abundance of peroxisome proliferator-activated receptor gamma (*Pparγ*), a nuclear receptor that regulates the differentiation of mesenchymal stem cells to the adipocyte versus osteoblast lineage [16], was significantly elevated (*p* = 0.001) in the DP group at 4 weeks (Fig. 4a). Runt-related transcription factor 2 (*Runx2*), a transcription factor necessary for the development of immature osteoblasts from mesenchymal progenitor cells [17], tended to be lower (*p* = 0.08) in the DP group at 4 weeks compared to control (Fig. 4b). Additionally, relative abundance of osterix (*Osx*, *p* < 0.001), a downstream target of *Runx2* [17], was significantly lower after 4 weeks of DP supplementation compared to control (Fig. 4c). Bone morphogenetic protein 2 (*Bmp2*), an activator of *Runx2* [18], tended to be decreased (*p* = 0.07; Fig. 4d) and *Bmp4*, which plays a regulatory role in chondrogenesis [19] and a potential precursor to future bone formation [20], was significantly decreased (*p* = 0.02) (Fig. 4e). There were no differences in gene expression of these osteoblast differentiation markers between treatment groups at 12 weeks.

Suppression of genes associated with osteoblast activity at 4 weeks in the DP group was also observed. Alkaline phosphatase (*Alp*) (*p* = 0.01) which is considered an early marker of osteoblast activity [21] and the extracellular matrix proteins, collagen type 1, alpha 1 (*Col1a1*) and osteocalcin (*Bglap2*), were significantly reduced (*p* < 0.01) in the DP group compared to control at 4 weeks (Fig. 4f–h). However, no differences in *Alp*, *Col1a1*, and *Bglap2* were observed between the DP and control groups after 12 weeks of treatment.

**Fig. 5:**
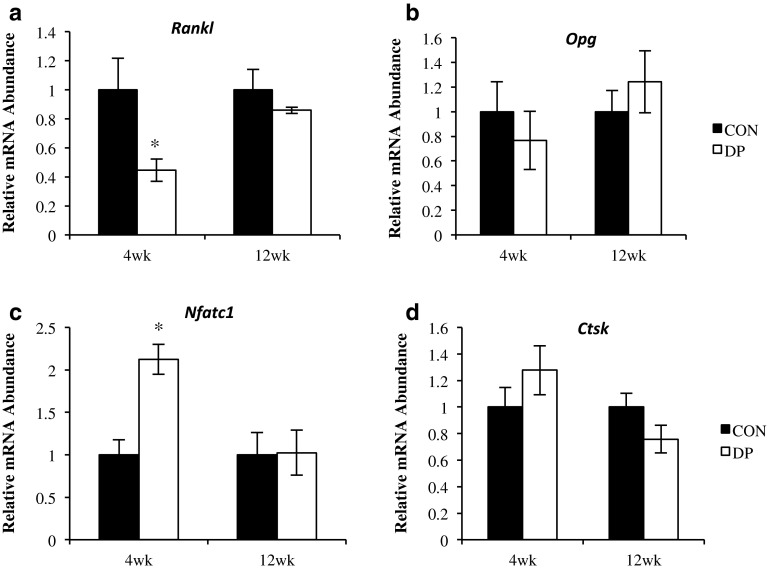
Alterations in gene expression related to osteoclast differentiation and activity. The relative mRNA abundance of **a** receptor activator of NF-κB ligand (*Rankl*) and **b** osteoprotegerin (*Opg*) in the flushed femur, and **c** nuclear factor of activated T cells (*Nfatc1*), and **d** cathepsin K (*Ctsk*) in the bone marrow are shown after 4 and 12 weeks of DP supplementation. *DP treatment group is different from control at given time point, *p* < 0.05; *n* = 9–10 mice/group

Osteoclast differentiation and activity were also affected by DP supplementation. Expression of receptor activator of NF-κB ligand (*Rankl*) was suppressed in the DP group, suggesting a decrease in osteoclast differentiation (Fig. 5a). In contrast, nuclear factor of activated T cells (*Nfatc1*), which is a considered a major regulator of RANKL-induced osteoclast differentiation [22], was up-regulated at 4 weeks in the DP group compared to control (Fig. 5c). Although this response was not anticipated, it could represent an attempt to compensate for the decrease in osteoclasts that had occurred or could be tied to *Nfatc1*s role in osteoblast differentiation. As an indicator of osteoclast activity, the relative expression of cathepsin K (*Ctsk*), a protease essential to matrix solubilization found abundantly in osteoclasts [23], was not different between treatment groups at either time point (Fig. 5d).

## Discussion

Previously, we have reported that 24 weeks of dietary supplementation with DP resulted in a substantial increase (i.e., 30–40 %) in trabecular bone mass in aging animals [15]. These positive effects on bone occurred primarily during the first 12 weeks of supplementation with DP at 25 % of the diet (w/w). The current study was designed to investigate the early bone metabolic changes occurring in response to DP in a model of age-related osteoporosis. The findings of this study demonstrated for the first time that the improved bone mass and microarchitecture were consequence of an initial suppression of bone turnover at 4 weeks, followed by normalization of bone remodeling at least at some anatomical sites after 12 weeks. In the distal femur metaphysis, osteoblast and osteoclast surfaces were decreased at the initial time point. These changes coincided with a reduction in the bone formation rate and mineralizing surface in the distal femur and a decrease in plasma P1NP, a systemic indicator of bone formation. Interestingly, improvements in bone density (whole body and spine) as well as trabecular bone volume and cortical thickness occurred at some sites by the end of 4 weeks despite these metabolic changes. Although the possibility that DP had an earlier effect on bone formation and/or resorption that resulted in these improvements in trabecular and cortical bone cannot be ruled out, these data could suggest that the metabolic response to DP is similar to that observed with antiresorptive therapies. Increases in BMD have been reported with bisphosphonates that disproportionately suppress the activity of osteoclasts over that of osteoblasts [24]. It is not clear if this was the case with DP because of the sizable (i.e., 80 %) decrease in the rate of bone formation and the inability to directly measure the rate of bone resorption using histomorphometry [25].

By the end of 12 weeks, DP supplementation had a very different effect on bone metabolism than the response observed at the earlier time point. The osteoblast and osteoclast surfaces of the DP supplemented animals had normalized to the level of the controls within the cancellous bone of the distal femur. Interestingly, the bone formation rate and mineralization surface increased by 54 and 69 %, respectively, in the DP groups compared to the controls, but these changes did not reach the level of statistical significance. Inherent variability in double labeling for histomorphometric analyses may have contributed to this lack of a statistically significant response [26]. These data can lead one to conclude that a biphasic response occurred in the distal femur metaphysis characterized by an initial suppression of bone turnover followed by normalization or perhaps even an increase in the activity of the existing osteoblast on the surface of the cancellous bone. Hooshmand et al. [14] reported transient changes in bone metabolism in postmenopausal women consuming DP (100 g/day) over a 1-year period. They also concluded that DP inhibited postmenopausal bone loss by suppressing the accelerated rate of bone turnover associated with ovarian hormone deficiency [14]. In contrast to the rate of bone turnover associated with ovarian hormone deficiency, age-related bone loss results from a decline in the rate of bone turnover [5]. However, in this study, the serum indicators of bone formation and resorption (i.e., P1NP and PYD) continued to be suppressed at 12 weeks despite the fact that osteoblast and osteoclast surfaces had normalized. When these systemic biomarkers of bone metabolism are considered in conjunction with the histomorphometry data at the distal femoral metaphysis, they suggest that a site-specific response to DP over time could contribute to the discrepancy between the histomorphometric data focused on the distal femur metaphysis and the systemic markers.

Analysis of gene expression provided insight into DPs effect on transcriptional regulation of osteoblasts differentiation and activity. Early regulators of mesenchymal cell allocation to the osteoblast lineage include BMP2 and BMP4, which are members of the transforming growth factor β superfamily of proteins. These BMPs were originally identified as requirements for embryonic skeletal development, but were later recognized for playing a critical role in osteoblast differentiation and bone formation in adult bone [27]. Our findings demonstrated that Bmp4 was significantly suppressed in response to DP compared to animals on the control diet. BMP2 and BMP4 have been shown to act through the type I receptor to induce *Runx2* and its downstream target, Osterix [27, 28]. Although the relative abundance of *Bmp2* and *Runx2* in bone was not altered significantly with DP in this study, the reduction in osteoblasts coincided with the down-regulation of *Osx. Osx* encodes for the transcription factor, Osterix, which is required for osteoblast formation [28]. Coincident with the down-regulation of these key mediators of osteoblastogenesis, the relative expression of PPARγ was increased (>1.6-fold). PPARγ is known to direct pluripotent mesenchymal stem cells toward the adipocyte lineage as opposed to osteoblasts or other cells types [16]. Thus, it is conceivable that the bioactive components in DP are driving key regulators of osteoblast and adipocyte cell lineage at 4 weeks away from the osteoblast lineage. In contrast to the initial inhibition of osteoblast differentiation and activity that occurred in this study, by the end of 12th week of DP treatment, the osteoblast surface had returned to the level of the control animals and there were no significant differences in the BMPs *Runx2* or *Osx* at the transcriptional level.

In conjunction with the decrease in osteoblast surface at the initial time point, suppression of indicators of osteoblast activity was also observed using dynamic bone histomorphometry. The relative abundance of *Alp*, which encodes for alkaline phosphatase and which is considered an indicator of osteoblast cellular activity or number, was suppressed. Likewise, the expression of *Col1a1* and *Bglap2* was lower in the DP treated animals. These findings would indicate that type I collagen which provides the major extracellular protein structure in bone and osteocalcin, a noncollagenous protein secreted in the later stages of osteoblast differentiation to facilitate the formation of hydroxyapatite, would be down-regulated at the early time point [29]. Taken together, these alterations in genes encoding for proteins associated with osteoblast activity are consistent with the observed decrease in osteoblasts and their potential to secrete protein matrix occurring in the DP group when compared to controls at 4 weeks. However, these responses were temporary, and by the 12-week time point, there was no indication of transcriptional alterations associated with osteoblast activity.

Factors involved in regulating osteoclastogenesis and indicators of osteoclast activity were also investigated to determine the mechanism through which osteoclasts were decreased in the distal femoral metaphysis after 4 weeks of DP supplementation. *Rankl*, a key differentiation factor for osteoclastogenesis expressed by osteoblasts [30], was suppressed in the DP group compared to control at 4 weeks, and there was no alteration in *Opg* expression. These findings are in line with our previous report [10] demonstrating DP suppressed bone resorption by down-regulating gene expression and proteins levels of RANKL in bone of gonadal hormone-deficient male rats. However, in the current study, the relative abundance of *Nfatc1* was unexpectedly increased at 4 weeks in the DP group compared to the control group. We have previously shown in rodent models of ovarian hormone deficiency that DP down-regulated *Nfatc1* [12]. Whether or not this was indicative of a compensatory response to the reduction in osteoclasts is unclear. A contributing factor may have been the alterations in BMP4. Aside from its role in osteogenesis, BMP4 is also considered a regulator of hematopoietic stem cell, HSC [i.e., c-kit^+^, Sca-1^+^, Lin^−^ (KSL) cells], function and number [31]. However, further studies are warranted to determine whether a disturbance in the HSC population induced by DP over the first 4 weeks contributed to the phenomenon. With regard to osteoclast activity, no changes in the relative levels of *Ctsk* were demonstrated at 4 or 12 weeks. Similar to the observation with osteoblasts, by the 12th week of the study, all of the regulators of osteoclastogenesis were restored to the level of control animals, which is consistent with the osteoclast surface histomorphometry data.

Other functional foods that are rich in phenolic compounds have been shown to have bone protective properties [32–35]. Soy protein and its isoflavones have been one of the most studied functional foods in relation to bone health. Soy prevents bone loss in young growing animals and animal models of gonadal hormone deficiency by suppressing bone resorption and inducing a modest increase in genes associated with osteoblast differentiation [32, 36, 37]. Other botanical products such as blueberries and green tea polyphenols have also been shown to protect against bone loss in animal models of osteoporosis [33–35, 38]. Many of these plant-based foods are known to have bioactive components with potent antioxidant properties [33, 37–39]. In the current study, we demonstrated that by 12 weeks, DP had increased circulating glutathione peroxidase. DP is known to have a high oxygen radical absorbance capacity score, attributed in large part to it phenolic compounds [40]. Reactive oxygen species are known to have negative effects on bone metabolism by down-regulating osteoblast activity and up-regulating osteoclast activity [41]. Antioxidants, such as glutathione peroxidase, are produced by a number of cells, including osteoblasts [42]. It remains to be determined if the increase in glutathione peroxidase observed in this study is involved in mediating the secondary response occurring at 12 weeks that results in improved bone mass.

The results of these studies show that the osteoprotective effects of DP in an animal model of age-related bone loss are associated with an initial suppression of bone turnover followed by a secondary metabolic phase that may favor enhanced bone formation. This secondary response to DP coincided with an increase in systemic glutathione peroxidase, suggesting that the effects of DP may be mediated through antioxidant mechanisms. In agreement with our previous reports [9–11], we expected that osteoclast activity would be suppressed; however, the suppression of osteoblast activity at the early time point was not anticipated. To our knowledge, this study is the first to examine the short-term bone metabolic response to DP in an aging model and provides new insights into the mechanisms through which bone is altered. Further studies are needed to determine the role of glutathione peroxidase in the secondary metabolic phase induced by DP and the bioactive components of DP that are exerting these osteoprotective effects.
